# Molecular basis of arrhythmic substrate in ageing murine peroxisome proliferator-activated receptor γ co-activator deficient hearts modelling mitochondrial dysfunction

**DOI:** 10.1042/BSR20190403

**Published:** 2019-12-13

**Authors:** Charlotte E. Edling, Ibrahim T. Fazmin, Khalil Saadeh, Karan R. Chadda, Shiraz Ahmad, Haseeb Valli, Christopher L.-H. Huang, Kamalan Jeevaratnam

**Affiliations:** 1 Faculty of Health and Medical Sciences, University of Surrey, Guildford GU2 7AL, United Kingdom; 2 Physiological Laboratory, University of Cambridge, Downing Street, Cambridge CB2 3EG, United Kingdom; 3 Department of Biochemistry, Hopkins Building, University of Cambridge, Cambridge CB2 1QW, United Kingdom

**Keywords:** cardiac arrhythmia, connexins, voltage-gated channels

## Abstract

*Introduction*: Ageing and chronic metabolic disorders are associated with mitochondrial dysfunction and cardiac pro-arrhythmic phenotypes which were recently attributed to slowed atrial and ventricular action potential (AP) conduction in peroxisome proliferator-activated receptor γ co-activator deficient (*Pgc-1β*^−/−^) mice. *Methods*: We compared expression levels of voltage-gated Na^+^ channel (Na_V_1.5) and gap junction channels, Connexins 40 and 43 (Cx40 and Cx43) in the hearts of young and old, and wild-type (WT) and *Pgc-1β*^−/−^ mice. This employed Western blotting (WB) for Na_V_1.5, Cx40 and Cx43 in atrial/ventricular tissue lysates, and immunofluorescence (IF) from Cx43 was explored in tissue sections. Results were analysed using two-way analysis of variance (ANOVA) for independent/interacting effects of age and genotype. *Results*: In atria, increased age and *Pgc-1β*^−/−^ genotype each independently decreased both Cx40 and Cx43 expression without interacting effects. In IF experiments, both age and *Pgc-1β* deletion independently reduced Cx43 expression. In ventricles, age and genotype exerted interacting effects in WB studies of Na_V_1.5 expression. Young *Pgc-1β*^−/−^ then showed greater Na_V_1.5 expression than young WT ventricles. However, neither age nor *Pgc-1β* deletion affected Cx43 expression, independently or through interacting effects in both WB and IF studies. *Conclusion*: **Similar** pro-arrhythmic atrial/ventricular phenotypes arise in aged/*Pgc-1β^−/−^* from **differing** contributions of altered protein expression and functional effects that may arise from multiple acute mechanisms.

## Introduction

Both increased age and metabolic disorders, the latter themselves age-dependent, and associated with physical inactivity [1], obesity [2], diabetes mellitus [3] and metabolic syndrome [4] constitute cardiac health issues of increasing clinical importance. In addition, strong observational and experimental evidence also suggests links between increased age and mitochondrial dysfunction. Thus, human ageing was associated with acquired damage to mitochondrial DNA (mtDNA), clonal expansion of these somatic mutations, and respiratory chain deficiency [5–7]. An autopsy study revealed increased frequencies of defects in the terminal enzyme of cardiomyocyte respiratory chain cytochrome-*c*-oxidase with age [8]. Age-associated mtDNA damage and compromised respiratory chain function was also reported in various other mammalian, including mouse [9], rat [10] and rhesus monkey [11], species. Conversely, experimental studies in the mouse-related genetic defects increasing levels of somatic mtDNA mutations to a premature ageing syndrome [7,12]. There is also evidence linking metabolic disorders with mitochondrial dysfunction. Analysis of atrial tissue obtained from diabetic patients during coronary artery bypass graft surgery suggested a complex I electron transport chain (ETC) abnormality and decreased state 3 respiration in cardiac mitochondria [13]. Similarly, obese mice fed a high-fat diet showed increased mitochondrial dysfunction, also associated with defective complex 1 [14].

Mitochondrial dysfunction in turn is associated with increased risks of cardiac arrhythmias which themselves represent a major source of clinical morbidity and mortality. Atrial fibrillation (AF) affects 1–3% of the developed world population [15–17]. Chronic AF increases risks of morbidity in the form of stroke [18] and of all-cause mortality [19–21]. Ventricular arrhythmias including ventricular fibrillation can cause sudden cardiac death, which accounts for 180000–200000 fatalities/year in the United States [22]. Mitochondria from chronic AF patients showed increased DNA damage [23,24], structural abnormalities [25] and impaired function [23,26]. Inherited mitochondrial disorders such as Kearns–Sayre syndrome predispose to fatal ventricular arrhythmias [27]. Similarly, experimental evidence also implicates mitochondrial dysfunction in arrhythmogenesis. Both acute and chronic mitochondrial dysfunction promote cardiac arrhythmogenesis in animal models [28]. Acute mitochondrial impairment with ischaemia–reperfusion was pro-arrhythmic in a guinea pig model which showed a ventricular fibrillation that could be suppressed by pharmacological manipulations of mitochondrial membrane potential [29,30]. Abnormal mitochondrial structure and function were also reported in goat and dog AF models [31,32].

Recent experimental studies have explored metabolic and cardiovascular alterations associated with mitochondrial deficiency using peroxisome-proliferator activated receptor γ (PPARγ) co-activator-1 (Pgc-1) deficient (*Pgc1^−/−^*) murine models. Pgc-1 co-activators constitute one of a number of transcriptional co-activators up-regulating catabolic processes acting on cellular energy stores in response to external stressors that result in increased energy demand that include sustained exercise or fasting. Of the three Pgc-1 family members, *Pgc-1α* and *Pgc-1β* are highly expressed in tissues with high energy requirements and mitochondrial content including heart, skeletal muscle, kidney and brown adipose tissue. In contrast, Pgc-1-related co-activator (PRC) is ubiquitously expressed across different tissues. *Pgc-1α* expression is up-regulated by physiological stimuli such as fasting, exercise and cold temperatures, adapting tissues to conditions of high energy demand. In contrast, *Pgc-1β* expression is not altered by these stimuli, consistent with roles in maintaining basal mitochondrial function [33]. Recent experimental studies reported that the *Pgc-1β*^−/−^ murine model shows reduced cardiac mitochondrial volume fractions and expression of ETC genes, but normal internal mitochondrial structure with no changes in cristae surface density per unit mitochondrial volume [34].

Subsequent reports then described electrophysiological and Ca^2+^ homoeostatic changes [35] extending to increased incidences of extrasystolically provoked atrial and ventricular arrhythmias that could then be attributable to slowed conduction velocity (*θ*) in the *Pgc-1β^−/−^* murine model [36–39]. The latter could only be accounted for by a combination of alterations associated both with inward Na^+^ current activation [40] and passive resistance to longitudinal current flow [36,38,41]. Thus, reduced maximal action potential (AP) upstroke rates (d*V*/d*t*)_max_ and Na^+^ current activation typically mediated by the cardiac Na^+^ channel, voltage-gated Na^+^ channel (Na_V_1.5), only partially accounted for the change in conduction velocity. However, fibrotic changes also observed could additionally compromise gap-junction mediated passive longitudinal current flow between cardiomyocytes, typically carried by connexins (Cx) [42]. Of the four main murine Cx30.2, Cx40, Cx43 and Cx45 connexin isoforms, Cx40 occurs in the intercalated discs of atrial myocytes and Cx43 occurs in the intercalated discs of both atrial and ventricular myocytes [43,44].

However, the relative contributions of altered expression in the underlying Na_V_1.5 and connexin 40 and 43 (Cx40 and Cx43) proteins, and of modifications in their properties to the above phenotypic changes accompanying ageing and mitochondrial dysfunction is unknown. The present experiments accordingly examined expression levels of these molecules using Western blotting (WB) and immunofluorescent quantification on cardiac tissue lysates or sections, respectively. To facilitate comparisons with the previous electrophysiological investigations [36–38,41], the studies were performed in both young and aged, and in both atria and ventricles of both wild-type (WT) and *Pgc-1β*^−/−^ hearts.

## Materials and methods

The studies conformed to the Animals (Scientific Procedures) Act 1986 Amendment Regulations 2012 and ethical approval by the University of Cambridge Animal Welfare and Ethical Review Body (AWERB). Briefly, homozygous *Pgc-1β*^−/−^ mice were obtained by crossing heterozygous *Pgc-1β*^−/+^ mice derived from a triple LoxP targeting system which was used to excise exons 4 and 5 of the *Pgc-1β* gene when crossing with Cre-expressing mice [34,35]. WT and *Pgc-1β*^−/−^ C57/B6 mice were bred. They were housed in plastic cages in a temperature-controlled (21°C) room, with a 12-h light–dark cycle and free access to water, sterile chow (RM3 Maintenance Diet; SDS, Witham, Essex, U.K.), bedding and environmental stimuli. Four experimental groups of young (aged between 18 and 19 weeks) and aged (>70 weeks), and WT and *Pgc-1β*^−/−^ yielded a 2 × 2 factorial study design. The mice were killed by cervical dislocation, their hearts excised and atria and ventricles separated for storage at −80°C for WB analysis, or fixed in formalin for paraffin embedding for immunofluorescence (IF) studies.

### Antibody reagents

WB studies used monoclonal primary antibody (dilution 1:500) to Na_V_1.5 raised in rabbits to endogenous Na_V_1.5 protein and its (190 and 135 kDa) degradation products (D9J7S #14421; Cell Signaling Technology, Leiden, The Netherlands). WB and IF studies used polyclonal anti-Cx40 (at dilution of 1:500 for WB) raised in goat to an epitope near the C-terminus of human Cx40 (sc-20466; Santa Cruz Biotechnology, Inc. Heidelberg, Germany) and polyclonal anti-Cx43 (at a dilution of 1:1000 for both WB and IF) raised in rabbit to the C-terminal segment of the cytoplasmic domain (amino acids 363–382 with N-terminally added lysine) of human/rat Cx43 (C6219: Sigma–Aldrich, Dorset, U.K.). Polyclonal anti-glyceraldehyde 3-phosphate dehydrogenase (GAPDH) (dilution 1:1000) was raised in rabbits to full-length native (purified) human GAPDH (ab9485: Abcam, Cambridge, U.K.). The WB studies used secondary donkey IgGs against goat (IRDye™ 800CW and 680RD; peak excitation/emission wavelengths 778/794 and 680/694 nm, respectively; dilution 1:15000) for blots of Cx40 and donkey IgGs against rabbit (IRDye™ 680RD; peak excitation/emission wavelengths 680/694 nm; dilution 1:10000) for blots of Na_V_1.5, Cx43 and GAPDH (LI-COR Biosciences, Cambridge, U.K.). The IF studies used secondary anti-rabbit IgG (Alexa Fluor 568; peak excitation/emission wavelengths 578/603 nm; dilution 1:250) raised in goats (Abcam, Cambridge, U.K.).

### WB analysis of Na_V_1.5, Cx40 and Cx43 expression

Protein expression was quantified using sodium dodecyl sulphate/polyacrylamide gel electrophoresis (SDS/PAGE) and WB [45] on tissue homogenates prepared from mouse atrial and ventricular samples. For protein extraction, the samples were weighed, placed on ice, chopped into small pieces by scalpel, placed in 450 µl lysis buffer (150 mM NaCl, 25 mM tris(hydroxymethyl)aminomethane (tris), pH 7–8, 1% Triton-X100 detergent, 5 mM ethylenediaminetetraacetic acid (EDTA) and Roche® cOmplete™ mini protease inhibitor (Merck KGaA, Germany) and homogenised (Stuart® Tissue Homogeniser, Cole-Parmer, U.K.). For the ventricular samples, a further 450 µl lysis buffer was then added. The mixture was left shaking on ice for 60 min, vortexing at 0, 30 and 60 min. Samples were then separated by centrifugation at 12000 rpm for 20 min, following which the supernatant containing clear lysate was transferred into Eppendorf tubes and the pellet discarded. The lysate was assayed for protein content by bicinchoninic acid (BCA) assay (Thermo Scientific Microplate BCA Protein Assay Kit #23252: manufacturer-recommended protocol) [46,47]. For SDS/PAGE, the samples were incubated with a loading buffer (12.8 ml tris, pH 6.8, 3.2 g sodium dodecyl sulphate (SDS), 1.85 g dithiothreitol (DTT), 16 ml 100% glycerol, Bromophenol Blue, 11.2 ml H_2_O) in the ratio of 3:1 volume of clear lysate to loading buffer and warmed for 5 min at 70°C.

Samples were then loaded into the wells of a Mini-Protean TGX™ (Bio-Rad, U.K.), 4–15% acrylamide gradient, precast gel. For obtaining Na_V_1.5 blots, 20 µg were loaded per lane and for Cx40 and Cx43, 30 µg were loaded per lane. Samples were run and compared against a housekeeping protein (GAPDH) as a loading control. The running buffer used was tris-glycine-SDS running buffer containing 25 mM tris, 192 mM glycine, 0.1% SDS, pH 8.3 following dilution to 1× concentration with water (Bio-Rad, U.K.). A coloured protein ladder (Precision Plus Protein™ Dual Colour Standards, Bio-Rad, U.K.) was used to estimate molecular weights of protein bands. The gels were loaded into an electrophoresis tank connected to a power pack (Bio-Rad, U.K.). The electrophoresis tank was immersed in an ice bath and the gel exposed to a potential of 120 V for 30 min followed by 250 V for 20 min.

Proteins were electrophoretically transferred on to polyvinylidene fluoride (PVDF) membranes (Immobilon™ PVDF membrane, Merck KGaA, Germany) activated by 30 s immersion in methanol followed by equilibration in transfer buffer (proprietary Trans-Blot® Turbo™ Transfer Buffer, Bio-Rad, U.K.). Semi-dry transfer used a Trans-Blot® Turbo™ kit (Bio-Rad, U.K.). A stack of six sheets of filter paper (Trans-Blot® Turbo™ mini-stack, Bio-Rad, U.K.) was placed on the bottom cassette positive electrode. Upon this PVDF membrane, the acrylamide gel and a further six-sheet filter paper stack were placed, before placing the stack between an upper cassette negative electrode and the bottom cassette positive electrode, placing the cassette placed into a Trans-Blot® Turbo™ (Bio-Rad, U.K.). The transfer occurred using 1.3 A current and 25 V potential for 10 min of transfer time. The membranes were blocked with Odyssey® blocking buffer (LI-COR Biosciences, EU) for 1 h at room temperature on an orbital shaker and the membrane rinsed with PBS-T (0.1% Tween) and incubated with primary antibody diluted in Odyssey® blocking buffer diluted 33% in PBS-T, overnight at 4°C, on an orbital shaker.

After overnight incubation with the primary antibodies, the membrane was washed for 10 min in PBS-T and washed again twice. Secondary antibodies conjugated with dyes for near infrared fluorescence (NIF) with absorbance and emission spectra in the range of 600–800 nm were used. Secondary antibodies were diluted in Odyssey® blocking buffer diluted 33% in PBS-T and incubated with the membrane at room temperature for 45 min on an orbital shaker. Stripping was employed to re-probe membranes with multiple antibodies. Membranes were stripped by using NewBlot™ IR stripping buffer (LI-COR Biosciences, EU) using the manufacturer’s protocol. Briefly, the stripping buffer was diluted to 1× dilution by mixing one part buffer with four parts water. The blot was incubated with this buffer for 15 min at room temperature on an orbital shaker. Incubation was followed by washing with wash buffer for 5 min, repeating the wash twice, proceeding to the blocking step as mentioned above and probing with the other antibodies.

Blots were imaged with an Odyssey® Fc imaging system (LI-COR Biosciences, EU) exciting the blots with NIF excitation and measuring emission from the secondary antibodies at 600 and 800 nm. This allowed the use of two secondary antibodies on one blot, with one image taken with a 600-nm filter and another with an 800-nm filter. The molecular weight of protein bands was estimated by comparing with the pre-stained protein ladder. Protein quantification measured intensities using Image Studio™ software (LI-COR Biosciences, EU). Background signal was defined as the median pixel intensity of a defined region surrounding the protein band, and this median value multiplied by the area of the protein band quantification box was subtracted from the total signal from the protein band to give the corrected signal for a particular protein. Relative expression of Na_V_1.5, Cx40 and Cx43 was quantified by first obtaining the ratio of corrected target protein signal to GAPDH signal. This was then converted into a relative value for groups of atria or ventricles (four groups of: young WT, old WT, young *Pgc-1β^−/−^* and old *Pgc-1β*^−/−^) by calculating the value of a corrected protein signal as a percentage of the sum of all four corrected protein signals across the four groups.

### IF staining

Sectioning of paraffin wax embedded atrial and ventricular samples into 4-μm (range 3–5 μm) thickness. Following mounting on glass slides, dewaxing, rehydration and permeabilisation (using 0.1% Triton X-100 detergent) using an automated stainer, heat-induced antigen retrieval at 95°C and a pH of 9.0, washes with Tris-buffered saline (TBS) and blocking with universal blocking buffer, slides were incubated with primary antibodies at 4°C overnight, washed with TBS, and were incubated for 30 min at room temperature with secondary antibodies conjugated with Alexa Fluor™ fluorescent dye (Thermo Fisher Scientific, U.S.A.). A Nikon® Intensilight mercury-fibre epifluorescence lamp was used to provide excitation light (Nikon Instruments Europe B.V., United Kingdom). Images of the fluorescently labelled slides used a Nikon® Eclipse-Ci microscope system and Nikon® DS-Fi2 camera (Nikon Instruments Europe B.V., United Kingdom) controlled by NIS-Elements Advanced Research software (Nikon Instruments Europe B.V., U.K.). Exposure settings were set using the following criteria: the longest possible exposure time before getting software-determined saturated pixels. The signal exposure time was set as 200 ms; 40× magnification was used. Between three and four images per slide were used to calculate average values per biological replicate.

Semi-quantitative analysis of the acquired images used ImageJ software (National Institutes of Health, U.S.A.), using a superimposed histomorphometric grid method as described previously in [48]. Images were split into three component channels representing red and green (red corresponding to Cx43). Once split into different channels, a grid was overlaid on each image. The size of the grid was kept constant at 10000 pixels per square of the grid. This allowed for approximately 475 individual squares to be placed over an image. An investigator analysed the images blindly. Expression levels of a protein were expressed as the number of squares containing fluorescent signal as a percentage of total tissue area.

### Statistical analysis

Data are expressed as mean values ± standard errors of the mean (SEM) unless otherwise stated. Statistical tests were performed using Statistical Package for the Social Sciences (SPSS) Statistics software (IBM, U.S.A.). *n* denotes the number of biological replicates (individual animals) in each group. Data were subjected to a test of normality (Shapiro–Wilk test) and a test of homoscedasticity (Levene’s test) before proceeding to two-way analysis of variance (ANOVA) to explore for significant independent and interacting effects of age and genotype, followed by post-hoc testing with Tukey’s honest significant difference (HSD)test for pairwise comparisons, both to a significance level of *P*<0.05.

## Results

The experiments explored the molecular basis of the previous electrophysiological findings (see ‘Introduction’ section) that associated ageing and the *Pgc-1β^−/−^* genotype with pro-arrhythmic reductions in (d*V*/d*t*)_max_ and conduction velocities (*θ*) in both the atria and the ventricles of murine hearts. The latter alterations in AP parameters were then attributed to a combination of reductions in both regenerative inward current, dependent upon Na^+^ channel activation, and reduced myocyte–myocyte coupling determining longitudinal current flow between successive cells, influenced by gap junction conductance. These electrophysiological features respectively depend upon atrial and ventricular, Na_V_1.5 (Na^+^ channel) and Cx40 and Cx43 (gap junction channel) expression and function [42,49]. We accordingly measured and compared expression levels of Na_V_1.5, Cx40 and Cx43 in atria and ventricles from four experimental, young and aged, WT and *Pgc-1β*^−/−^ mouse groups in a 2 × 2 factorial design, matching ose adopted in the previous electrophysiological studies statistically testing for independent or interacting effects on these of increased age and *Pgc-1β*^−/−^ genotype [36–38].

### Western blot assessments of atrial Na_V_1.5 expression levels

Obtaining quantified expression levels of these key proteins was first performed by WB using antibodies specific to Na_V_1.5, Cx40 and Cx43 on atrial or ventricular tissue lysates fractionated by SDS/PAGE, with at least five biological replicates per group. Results of densitometry of their Western blot digital images, and the intensities of target protein bands were compared with those of a loading GAPDH control. Complementary, independent, quantification of fluorescently immunolabelled stained images of tissue sections on at least four biological replicates per group counted areas of tissue showing fluorophore–conjugated secondary antibody fluorescence normalised to total tissue area, using a histomorphometric grid [48].

**Figure 1:**
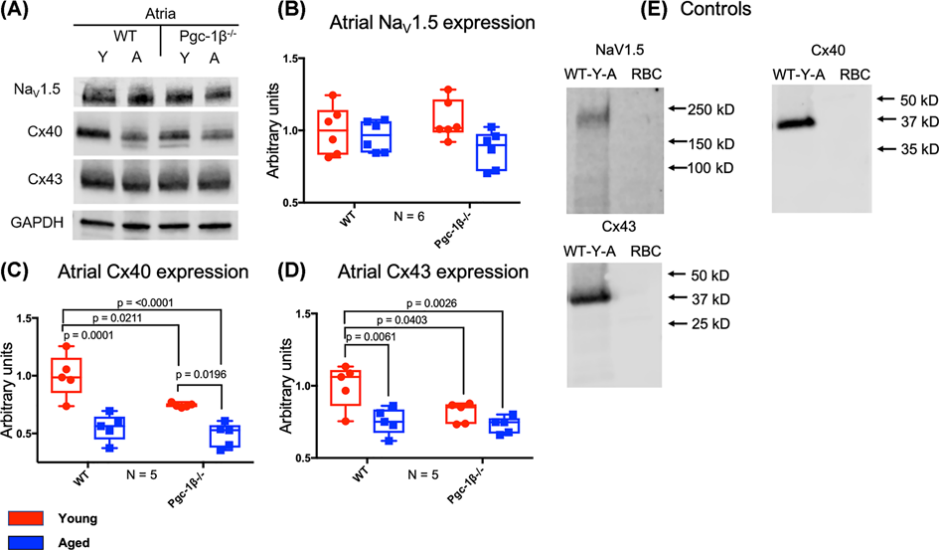
WB analysis of atrial Na_V_1.5, Cx40 and Cx43 expression (**A**) Representative Western blots of Na_V_1.5, Cx40, Cx43 and the housekeeping protein GAPDH, used as loading control. (**B**) Expression levels of atrial Na_V_1.5 obtained by densitometric analysis. (**C**) Expression levels of atrial Cx40 obtained by densitometric analysis. (**D**) Expression levels of atrial Cx43 expression obtained by densitometric analysis. (**E**) Control blots using lysed WT young atria or erythrocytes (RBC) together with the indicated primary and secondary antibodies. In (B–D), red boxes indicate young mice and blue boxes indicate aged mice. Primary monoclonal rabbit anti-Na_V_1.5 antibodies used at dilution 1:500; polyclonal goat anti-Cx40 used at dilution 1:500; polyclonal rabbit anti-Cx43 used at dilution 1:1000; polyclonal rabbit anti-GAPDH used at dilution 1:1000. Secondary donkey anti-goat IgG antibody used in blots staining for Cx40 at dilution 1:15000. Donkey anti-rabbit IgG antibody used at dilution 1:10000 in blots staining for Na_V_1.5, Cx43 and GAPDH. Significant *P*-values obtained by post-hoc testing with Tukey’s HSD test are indicated. Abbreviations: A, aged; N, number of biological replicates per experimental group; RBC, erythrocyte; WT-Y-A, wild type young atrial.

Increased age and *Pgc-1β^−/−^* genotype exerted markedly different effects on Na_V_1.5 and connexin expression in atria and ventricles, suggesting contrasting contributions from remodelling of protein expression and functional changes in Na_V_1.5 and connexins. Thus, increased age and *Pgc-1β*^−/−^ genotype did not affect atrial Na_V_1.5 expression, but each factor independently reduced atrial connexin expression. Figure 1A shows Western blots of Na_V_1.5 obtained from atrial tissue lysates from young and aged, and WT and *Pgc-1β^−/−^* mice. Figure 1B summarises densitometrically derived Na_V_1.5 expression levels, for which two-way ANOVA suggested that increased age independently decreased atrial Na_V_1.5 expression (F = 4.81, *P*=0.040). There were no independent effects of genotype (F = 0.032, *P*=0.86), or significant effects from any interaction between age and genotype (F = 2.18, *P*=0.16) on atrial Na_V_1.5 expression. However, a post-hoc Tukey’s HSD test for multiple comparisons between experimental groups indicated that there were no significant differences in atrial Na_V_1.5 expression between any of the experimental groups.

### Western blot assessments of atrial Cx40 and Cx43 expression levels

Murine atrial myocytes express two major connexin isoforms, Cx40 and Cx43 [43,44,50]. Figure 1A shows Western blots of Cx40 obtained from atrial tissue lysates from young and aged, WT and *Pgc-1β*^−/−^ mice. Figure 1C summarises densitometric analyses of Cx40 expression levels. Increased age (F = 41.75, *P*<0.0001) and *Pgc-1β*^−/−^ genotype (F = 8.58, *P*=0.0098) independently decreased atrial Cx40 expression. However, there was no significant effect of interactions between age and genotype on atrial Cx40 expression (F = 3.027, *P*=0.10). Furthermore, post-hoc testing showed that old WT and young *Pgc-1β^−/−^* atria showed reduced Cx40 expression compared with young WT atria (*P*<0.0001, *P*=0.021, respectively) and old *Pgc-1β*^−/−^ atria showed reduced Cx40 expression compared with young *Pgc-1β^−/−^* atria (*P*=0.020). In the extreme case, old *Pgc-1β^−/−^* atria showed reduced Cx40 compared with young WT atria (*P*<0.0001).

Finally, Figure 1A,D shows Western blot results of Cx43 obtained from the atrial tissue lysates and the corresponding results of densitometric analysis from young and aged, WT and *Pgc-1β*^−/−^ mice. In common with patterns observed with atrial Cx40 expression, increased age (F = 13.96, *P*=0.0018) and *Pgc-1β*^−/−^ genotype (F = 5.79, *P*=0.029) independently decreased atrial Cx43 expression, with no interacting effects between age and genotype upon atrial Cx43 expression (F = 3.24, *P*=0.091). Furthermore, the post-hoc testing revealed that old WT and young *Pgc-1β*^−/−^ atria showed lower Cx43 expression than young WT atria (*P*=0.0061, *P*=0.040, respectively). In the extreme case, old *Pgc-1β^−/−^* atria showed reduced Cx43 expression compared with young WT atria (*P*=0.0026).

### IF assessment of atrial Cx43 expression level

**Figure 2:**
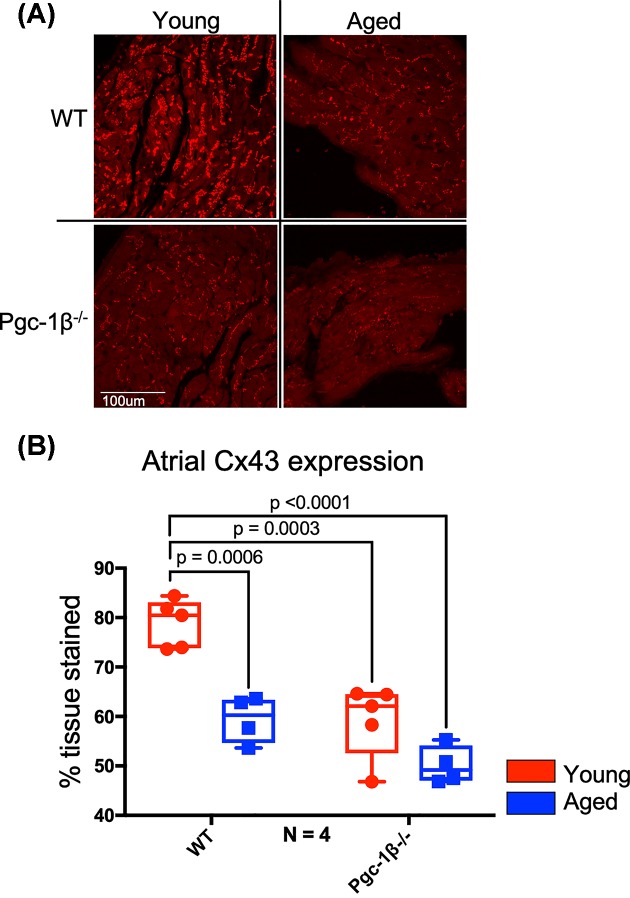
IF analysis of atrial Cx43 expression (**A**) Representative micrographs of Cx43 signal in stained atrial sections visualised at 40×. (**B**) Expression levels of atrial Cx43 as obtained by histomorphometric grid analysis. In (B), red boxes indicate young mice and blue boxes indicate aged mice. Primary polyclonal rabbit anti-Cx43 antibody used at dilution 1:1000; secondary goat anti-rabbit IgG antibody used at dilution 1:250. Significant *P*-values obtained by post-hoc testing with Tukey’s HSD tests are indicated. Note that brightness and contrast have been adjusted to make pictures more legible in print.

Complementary studies using IF staining could successfully be performed for atrial Cx43. Quantification of atrial Cx43 by IF staining closely agreed with the WB findings. Figure 2A,B shows representative images of atrial sections stained for Cx43 and the quantified atrial Cx43 expression levels. In common with the WB results, increased age and *Pgc-1β*^−/−^ genotype both independently reduced atrial Cx43 expression (F = 30.04, *P*<0.0001 and F = 30.84, *P*<0.0001, respectively), with no significant effects of interactions between age and genotype (F = 3.91, *P*=0.068). Subsequent post-hoc testing showed that aged WT, young *Pgc-1β^−/−^* and aged *Pgc-1β^−/−^* atria all showed decreased Cx43 expression compared with young WT atria (*P*=0.0006, *P*=0.0003, *P*<0.0001, respectively).

### Western blot assessments of ventricular Na_V_1.5 expression levels

**Figure 3:**
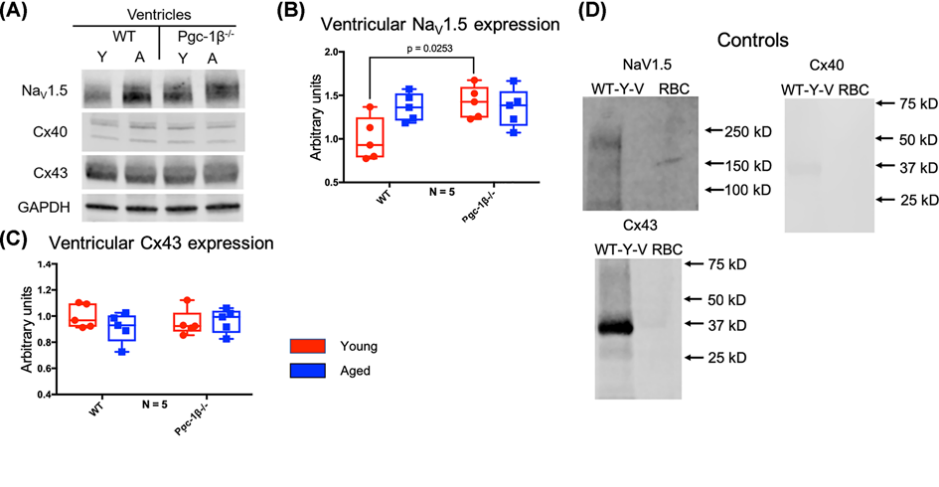
WB analysis of ventricular Na_V_1.5 and Cx43 expression (**A**) Representative Western blots of Na_V_1.5, Cx40, Cx43 and the housekeeping protein GAPDH, used as loading control. (**B**) Expression levels of ventricular Na_V_1.5 obtained by densitometric analysis. (**C**) Expression levels of ventricular Cx43 obtained by densitometric analysis. In (B,C), red boxes indicate young mice and blue boxes indicate aged mice. (**D**) Control blots using lysed WT young atria or erythrocytes (RBC) together with the indicated primary and secondary antibodies. Primary monoclonal rabbit anti-Na_V_1.5 antibody used at dilution 1:500; polyclonal goat anti-Cx40 antibody used at dilution 1:500; polyclonal rabbit anti-Cx43 used at dilution 1:1000; polyclonal rabbit anti-GAPDH used at dilution 1:1000. Secondary donkey anti-goat IgG antibody used at dilution 1:15000 in blots staining for Cx40, and donkey anti-rabbit IgG antibody used at dilution 1:10000 in blots staining for Na_V_1.5, Cx43 and GAPDH. Significant *P*-values obtained by post-hoc testing with Tukey’s HSD tests are indicated. Abbreviations: A, aged; N, number of biological replicates per experimental group; RBC, erythrocyte; WT-Y-V, wild type young ventricular.

Ventricular tissue gave results that contrasted with those obtained from atrial tissue. *Pgc-1β* deficiency then exerted a paradoxical effect of increasing ventricular Na_V_1.5 expression in contrast with the previous evidence for a compromised Na_V_1.5 function [36,41]. Figure 3A shows Western blots of Na_V_1.5 obtained from ventricular tissue lysates from young and aged, and WT and *Pgc-1β*^−/−^ mice. Densitometric quantifications shown in Figure 3B indicated that there were no significant independent effects of age on levels of Na_V_1.5 expression (F = 2.63, *P*=0.12). However, genotype exerted a significant independent effect on ventricular Na_V_1.5 expression (F = 4.90, *P*=0.042), and this was driven by a significant interacting effect of age and genotype on ventricular Na_V_1.5 expression (F = 5.41, *P*=0.034). Post-hoc testing subsequently demonstrated that ventricles from young *Pgc-1β^−/−^* animals showed increased Na_V_1.5 expression compared with those from young WT (*P*=0.025).

### Western blot and IF assessments of ventricular Cx40 and Cx43 expression levels

**Figure 4:**
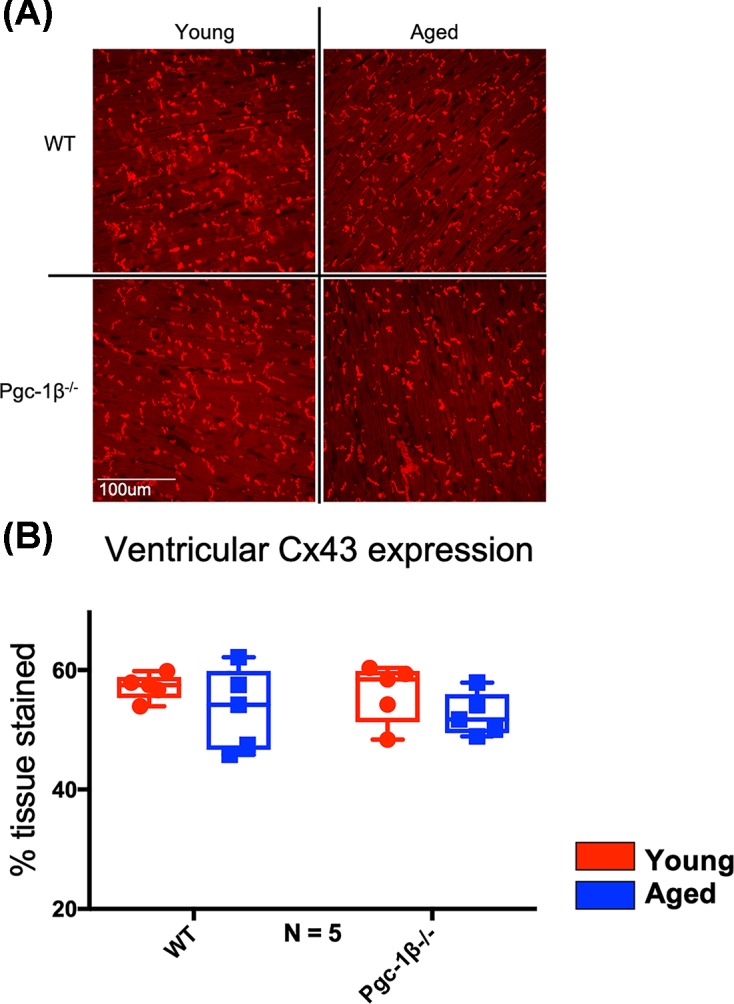
IF analysis of ventricular Cx43 expression (**A**) Representative micrographs of Cx43 signal in stained ventricular sections visualised at 40×. (**B**) Expression levels of ventricular Cx43 obtained by histomorphometric grid analysis. In (B), red boxes indicate young mice and blue boxes indicate aged mice. Primary polyclonal rabbit anti-Cx43 antibody used at dilution 1:1000; secondary goat anti-rabbit IgG antibody used at dilution 1:250. Significant *P*-values obtained by post-hoc testing with Tukey’s HSD tests are indicated. Note that brightness and contrast have been adjusted to make pictures more legible in print.

In contrast with the atrial results, age and genotype exerted neither independent nor interacting effects on ventricular connexin expression. Figure 3A shows Western blots of Cx40 obtained from ventricular tissue lysates from young and aged, and WT and *Pgc-1β^−/−^* mice. There was negligible ventricular Cx40 signal compared with the previously determined atrial Cx40 signal. This is expected: Cx40 is known not to occur in murine ventricular myocytes [43,44,50]. Thus, no attempt was made to perform densitometric analysis on ventricular Cx40. Cx43 is the predominant ventricular connexin [43,44,51]. Figure 3A also shows Western blots of Cx43 obtained from ventricular tissue lysates from young and aged, and WT and *Pgc-1β^−/−^* mice. Cx43 expression levels estimated from densitometric analysis (Figure 3C) demonstrated no significant independent effects of age (F = 0.64, *P*=0.43) or genotype (F = 2.432e-006, *P*=0.999), and no significant effect of the interaction between age and genotype (F = 1.13, *P*=0.26) on ventricular Cx43 expression. Similarly, Figure 4A,B shows representative IF images of ventricular sections stained for Cx43 and quantified ventricular Cx43 expression levels. As with the Western blot results, ventricular Cx43 expression was not affected by independent effects of age or genotype (F = 3.035, *P*=0.10, and F = 0.21, *P*=0.65, respectively) or an interaction between age and genotype (F = 0.00055, *P*=0.98).

## Discussion

**Figure 5:**
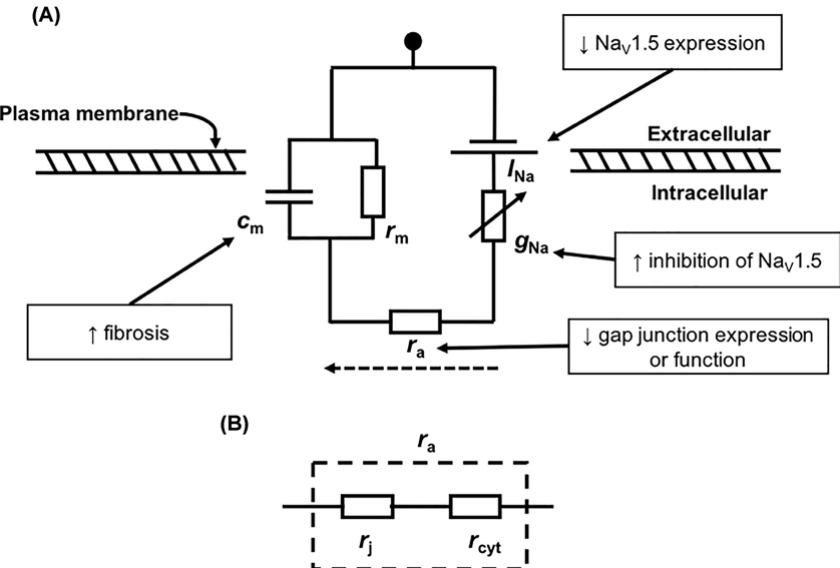
Proposed mechanistic links among Na_V_1.5 channels, gap junctions and conduction velocity (**A**) Circuit diagram schematic of the classical cable theory model of longitudinal conduction across cardiomyocytes. *I*_Na_ refers to the inward Na^+^ current that is carried by Na_V_1.5 channels, the conductance of which is represented by the term g_Na_. *r*_a_ refers to the longitudinal intercellular conductance along cardiomyocytes, which acts in the direction of AP propagation (dotted arrow). *c*_m_ and *r*_m_ refer to the capacitance and resistance of a quiescent region of adjacent membrane. In boxes, possible mechanisms that may reduce conduction velocity connected by arrow to the term of the cable equation which they affect. (**B**) The *r*_a_ term can be further broken down into two resistance values lying in series: *r*_j_, representing the resistance at gap junctions, and *r*_cyt_, the resistance within a cardiomyocyte arising due to the cytoplasm.

The present experiments were prompted by previous reports describing electrophysiological and anatomical, fibrotic, pro-arrhythmic changes resulting from ageing and the *Pgc-1β* knockout in C57/B6 mouse hearts. These studies associated age and *Pgc-1β*^−/−^ genotype with increased atrial and ventricular arrhythmic tendencies. They attributed these findings to reductions in (d*V*/d*t*)_max_ and AP conduction velocities, *θ*, despite normal AP durations and refractory periods [36,38,41]. Such abnormal reductions in excitation of regenerative activity are also associated with arrhythmic conditions such as the Brugada Syndrome and its murine replicates [50]. The reduced (d*V*/d*t*)_max_ observed in those earlier experiments was attributable to compromised Na^+^ channel function [42] through observations of reduced peak Na_V_1.5 currents in loose patch clamped young and aged *Pgc-1β^−/−^* compared with WT hearts *in situ* [40]. However, in both atria and ventricles of young and aged WT, the relationship of (1/*θ*) against (d*V*/d*t*)_max_ differed, while the corresponding plots from young and aged *Pgc-1β*^−/−^ were both similar to that of aged WT; the latter findings suggest a reduction in conductance of local intercellular current flow between cardiomyocytes. Local intercellular current in turn depends upon gap junction mediated myocyte–myocyte electrical coupling [42], dependent upon connexins (Cx). Of the four main murine Cx30.2, Cx40, Cx43 and Cx45 isoforms, Cx40 occurs in atrial and Cx43 occurs in both atrial and ventricular myocytes [43,44,50]. The relationships between various determinants of *θ* are presented in a summary diagram in Figure 5. Altered longitudinal resistances could then arise from decreased gap junction function potentially arising from the accompanying fibrotic changes.

These underlying functional alterations in otherwise normally expressed Na_V_1.5 and Cx40 and/or Cx43 function could arise directly from altered homoeostatic conditions associated with ageing or the *Pgc-1β^−/−^* genotype. Mitochondrial dysfunction increases reactive oxygen species (ROS) production up to ten-fold [52]. ROS decrease early Na^+^ current [53], modify Na^+^ and L-type Ca^2+^ channel inactivation kinetics, increase late Na^+^ current and oxidise RyR2 increasing SR Ca^2+^ leak thereby modulating intracellular Ca^2+^ cycling [54–56]. The associated increases in [NADH]_i_ also produced rapid onsets of dose-dependent (20–100 μM), persistent, approximately ≤50%, reductions in maximum Na^+^ current in HEK cells expressing human Na_V_1.5, despite unchanged activation and inactivation voltage dependences and mRNA and protein expression [57,58].

*Pgc-1β*^−/−^ cardiomyocytes also demonstrated altered Ca^2+^ homoeostasis evidenced in increased amplitudes of electrically evoked Ca^2+^ transients, extrasystolic Ca^2+^ release events associated with early and delayed after depolarisations, and propagated diastolic Ca^2+^ waves following such Ca^2+^ release events [35]. These could directly or indirectly modulate Na_V_1.5. Thus Ca^2+^ could directly bind to EF-hand intracellular motifs in Na_V_1.5, bind with EF-hand motifs on Ca^2+^-calmodulin (CaM) that in turn reacts with IQ sites on Na_V_1.5, or bind to CaM-dependent kinase II (CaMKII) activating its phosphorylating, inhibitory action on Na_V_1.5 [59,60]. Finally, recent reports demonstrated that both genetic modifications in RyR2 [59] and acute activation of protein kinase A (PKA)-independent exchange protein increase ryanodine receptor mediated sarcoplasmic reticular Ca^2+^ release [61] with reversible Na^+^ channel effects [62]. Similarly, altered Ca^2+^ homoeostasis elevating cytosolic [Ca^2+^] leads to gap junction closure reducing intercellular coupling [63–65]. Raised intracellular [Ca^2+^] may also activate a calcineurin-dependent Cx43 phosphorylation decreasing cell–cell coupling in guinea pig cardiomyocytes [66]. Increased intracellular [Ca^2+^] has been implicated in gap junction uncoupling following ischaemic injury in rabbit models [42].

Alternatively, mitochondrial dysfunction accompanying age and *Pgc-1β* deletion may down-regulate Na_V_1.5, Cx40 and Cx43 protein expression either through actions at the transcriptional or translational/trafficking level. ROS can decrease Na_V_1.5 transcription and the consequent channel expression. An alternative splicing then produces non-functional Na_V_1.5 with reduced Na^+^ current [67]. Similarly, altered Na_V_1.5 expression was observed in Western blot studies applied to *RyR2-P2328S* hearts exhibiting increased sarcoplasmic reticular Ca^2+^ release [60]. Additionally, elevated intracellular NADH activates protein kinase C, which inhibits Na^+^ current in the absence of altered mRNA levels, suggesting post-transcriptional effects [58,67]. ROS also reduce Cx43 trafficking and function [28,68,69]. ROS up-regulates the tyrosine kinase c-Src which through phosphorylation inhibits Cx43 function [67].

The experiments in the present study explored the extent to which altered molecular Na_V_1.5, Cx40 and Cx43 expression as opposed to functional effects might contribute to the observed pro-arrhythmic electrophysiological phenotypes in ageing and *Pgc-1β*^−/−^, atria and ventricles. WB and immunofluorescent quantification on both atrial and ventricular tissue lysates or sections compared protein expression levels in experimental groups of young and aged, and WT and *Pgc-1β*^−/−^ experimental groups in a 2 × 2 factorial design that paralleled those adopted in previous electrophysiological studies [36–38,41].

In the ***atria***, neither increased age nor *Pgc-1β* deficiency affected Na_V_1.5 protein levels whether independently or with interaction. This finding is consistent with prior studies of aged and Pgc-1*β* deficient mice that have shown no change in RNA levels of Scn5a, the gene coding for Na_V_1.5 [70]. However, both factors of age and genotype independently decreased atrial Cx40 and Cx43 expression levels. This could potentially contribute to a decreased coupling between cardiomyocytes and the previously observed pro-arrhythmic reduction in atrial conduction velocities [37,38]. Previous reports have associated AF in both animals and humans with abnormal Cx40 expression. Epicardial electrode array mapping studies have shown that Cx40^−/−^ mice are known to have increased atrial arrhythmogenicity, with reduced atrial AP conduction velocities [71]. Functional implications of the observed reductions in Cx43 expression may be more complex. Isolated murine Cx43^+/−^ hearts do not show reduced atrial AP conduction velocities or atrial arrhythmias even with application of aggressive electrical pacing protocols [51]. This comparison suggests that Cx40 expression is more critical to atrial conduction and arrhythmogenesis than that of Cx43. Furthermore, atrial conduction velocities may also depend on relative expression levels of Cx40 and Cx43. Progressively decreased Cx40 expression in cultured neonatal murine atrial myocytes paradoxically increased conduction velocities likely reflecting accompanying up-regulated Cx43 expression [43]. Finally, adult atria do not show the neonatal pattern of increased Cx43 expression at gap junctions upon Cx40 deletion [43]. Similarly, studies of tissue derived from human patients undergoing bypass surgery demonstrated negative correlations between both Cx40 expression levels and the proportion of Cx40 relative to total connexin (i.e. [Cx40]/[Cx43 + Cx40]) with measurements of atrial AP conduction velocity during sinus rhythm [44]. Imposition of 2 Hz electrical pacing then converted this relationship between proportional Cx40 expression and *θ* into a positive correlation [44].

In contrast, in ***ventricular*** myocardium, genotype but not age exerted independent effects, and these two factors additionally exerted interacting effects on Na_V_1.5 expression. Thus, young *Pgc-1β^−/−^* ventricles showed greater Na_V_1.5 expression than young WT. This contrasts with previous evidence for compromised Na_V_1.5 function, particularly reduced (d*V*/d*t*)_max_ suggested in the earlier electrophysiological experiments [36–38,40,41]. Additionally, gene transcription studies have shown Scn5a to not be affected by either age or *Pgc-1β* deficiency [72]. However, young and old, WT and *Pgc-1β^−/−^* ventricles did not show significant differences in expression of the corresponding Cx43 isoform. Together these findings exclude altered Na_V_1.5 protein expression as being major direct contributors to previous physiological findings reporting reduced atrial and ventricular Na_V_1.5 function [36–38,40,41]. They are compatible with alterations in connexin-mediated contributions of local intercellular current flow to conduction velocity arising from reduced atrial Cx40 and Cx43 but not ventricular Cx43 protein expression due to effects of age and *Pgc1β*^−/−^ genotype.

In both atria and ventricles, the unchanged level of NaV1.5 is unexpected even though consistent with the unchanged RNA level in both atrial and ventricular tissues [70,72]. This indicates the presence of other mechanisms than altered sodium current to explain the pro-arrhythmic features, for example Ca^2+^ homoeostasis as discussed.

## Conclusions

The findings of the present study therefore separate the roles of altered Na_V_1.5, Cx40 and Cx43 ***expression*** from changes in their ***function***, which compromise regenerative inward and local intercellular current generation. This subsequently may produce the pro-arrhythmic reductions in AP conduction seen in ageing or the *Pgc1β^−/−^* genotype. The findings of the present study contrast previously reported ***similar*** pro-arrhythmic electrophysiological atrial and ventricular, age and *Pgc-1β^−/−^* related phenotypes with ***differing*** respective contributions of altered protein expression, and accordingly differing functional effects on ion channels or gap junctions. These functional effects may arise due to the altered Ca^2+^ homoeostasis, oxidative stress or cellular redox potentials associated with the mitochondrial dysfunction accompanying age and/or *Pgc-1β* deletion [34,35,52–56].

## Abbreviations

(d*V*/d*t*)_max_: maximal action potential upstroke rate
AF: atrial fibrillation
ANOVA: analysis of variance
AP: action potential
Cx: connexin
Cx40: connexin 40
Cx43: connexin 43
ETC: electron transport chain
GAPDH: glyceraldehyde 3-phosphate dehydrogenase
HSD: honest significant difference
IF: immunofluorescence
mtDNA: mitochondrial DNA
Na_V_1.5: voltage-gated Na^+^ channel
NIF: near infrared fluorescence
Pgc-1: peroxisome proliferator-activated receptor γ co-activator-1
PVDF: polyvinylidene fluoride
ROS: reactive oxygen species
RYR2: Ryanodine receptor 2
SDS: sodium dodecyl sulphate
SDS/PAGE: SDS/polyacrylamide gel electrophoresis
SR: Sarcoplasmic Reticulum
TBS: Tris-buffered saline
WB: Western blotting
WT: wild-type
*θ*: conduction velocity

## References

[1] Mozaffarian D., Furberg C.D., Psaty B.M. and Siscovick D. (2008) Physical activity and incidence of atrial fibrillation in older adults: The Cardiovascular Health Study. Circulation 118, 800–807 10.1161/CIRCULATIONAHA.108.785626 18678768 PMC3133958

[2] Tedrow U.B., Conen D., Ridker P.M., Cook N.R., Koplan B.A., Manson J.A.E. et al. (2010) The long- and short-term impact of elevated body mass index on the risk of new atrial fibrillation. The WHS (Women’s Health Study). J. Am. Coll. Cardiol. 55, 2319–2327 10.1016/j.jacc.2010.02.029 20488302 PMC2880879

[3] Nichols G.A., Reinier K. and Chugh S.S. (2009) Independent contribution of diabetes to increased prevalence and incidence of atrial fibrillation. Diabetes Care 32, 1851–1856 10.2337/dc09-0939 19794003 PMC2752931

[4] Watanabe H., Tanabe N., Watanabe T., Darbar D., Roden D.M., Sasaki S. et al. (2008) Metabolic syndrome and risk of development of atrial fibrillation: The Niigata preventive medicine study. Circulation 117, 1255–1260 10.1161/CIRCULATIONAHA.107.744466 18285562 PMC2637133

[5] Ademowo O.S., Dias H.K.I., Burton D.G.A. and Griffiths H.R. (2017) Lipid (per) oxidation in mitochondria: an emerging target in the ageing process? Biogerontology 18, 859–879 10.1007/s10522-017-9710-z 28540446 PMC5684309

[6] Krishnan K.J., Greaves L.C., Reeve A.K. and Turnbull D. (2007) The ageing mitochondrial genome. Nucleic Acids Res. 35, 7399–7405 10.1093/nar/gkm635 17913753 PMC2190723

[7] Trifunovic A. and Larsson N.G. (2008) Mitochondrial dysfunction as a cause of ageing. J. Intern. Med. 167–178 10.1111/j.1365-2796.2007.01905.x 18226094

[8] Muller-Hocker J. and Müller-Höcker J. (1989) Cytochrome-c-oxidase deficient cardiomyocytes in the human heart-an age-related phenomenon. A histochemical ultracytochemical study. Am. J. Pathol. 134, 1167–1173, 2541614 PMC1879907

[9] Piko L., Hougham A.J., Bulpitt K.J., Pikó L., Hougham A.J. and Bulpitt K.J. (1988) Studies of sequence heterogeneity of mitochondrial DNA from rat and mouse tissues: evidence for an increased frequency of deletions/additions with aging. Mech. Ageing Dev. 43, 279–293, 10.1016/0047-6374(88)90037-1 2849701

[10] Wanagat J. (2001) Mitochondrial DNA deletion mutations colocalize with segmental electron transport system abnormalities, muscle fiber atrophy, fiber splitting, and oxidative damage in sarcopenia. FASEB J. 15, 322–332 10.1096/fj.00-0320com 11156948

[11] Schwarze S.R., Lee C.M., Chung S.S., Roecker E.B., Weindruch R. and Aiken J.M. (1995) High levels of mitochondrial DNA deletions in skeletal muscle of old rhesus monkeys. Mech. Ageing Dev. 83, 91–101 10.1016/0047-6374(95)01611-3 8569289

[12] Larsson N.-G. (2010) Somatic mitochondrial DNA mutations in mammalian aging. Annu. Rev. Biochem. 79, 683–706 10.1146/annurev-biochem-060408-093701 20350166

[13] Schrauwen-Hinderling V.B., Kooi M.E. and Schrauwen P. (2016) Mitochondrial function and diabetes: consequences for skeletal and cardiac muscle metabolism. Antioxid. Redox Signal. 24, 39–51 10.1089/ars.2015.6291 25808308

[14] Wang X., West J.A., Murray A.J. and Griffin J.L. (2015) Comprehensive metabolic profiling of age-related mitochondrial dysfunction in the high-fat-fed ob/ob mouse heart. J. Proteome Res. 14, 2849–2862 10.1021/acs.jproteome.5b00128 25985803

[15] DeWilde S., Carey I., Emmas C., Richards N. and Cook D. (2006) Trends in the prevalence of diagnosed atrial fibrillation, its treatment with anticoagulation and predictors of such treatment in UK primary care. Heart 92, 1064–1070 10.1136/hrt.2005.069492 16387813 PMC1861124

[16] Friberg L., Hammar N., Pettersson H. and Rosenqvist M. (2007) Increased mortality in paroxysmal atrial fibrillation: report from the Stockholm Cohort-Study of Atrial Fibrillation (SCAF). Eur. Heart J. 28, 2346–2353 10.1093/eurheartj/ehm308 17670754

[17] Majeed A. (2001) Trends in the prevalence and management of atrial fibrillation in general practice in England and Wales, 1994-1998: analysis of data from the general practice research database. Heart 86, 284–288 10.1136/heart.86.3.284 11514479 PMC1729916

[18] Wolf P.A., Dawber T.R., Thomas H.E. and Kannel W.B. (1978) Epidemiologic assessment of chronic atrial fibrillation and risk of stroke: The Fiamingham Study. Neurology 28, 973–973 10.1212/WNL.28.10.973 570666

[19] Benjamin E.J., Wolf P.A., D’Agostino R.B., Silbershatz H., Kannel W.B. and Levy D. (1998) Impact of atrial fibrillation on the risk of death: The Framingham Heart Study. Circulation 98, 946–952 10.1161/01.CIR.98.10.946 9737513

[20] Chamberlain A.M., Gersh B.J., Alonso A., Chen L.Y., Berardi C., Manemann S.M. et al. (2015) Decade-long trends in atrial fibrillation incidence and survival: a community study. Am. J. Med. 128, 260–267.e1 10.1016/j.amjmed.2014.10.030 25446299 PMC4340721

[21] Friberg L. and Bergfeldt L. (2013) Atrial fibrillation prevalence revisited. J. Intern. Med. 274, 461–468 10.1111/joim.12114 23879838

[22] Chugh S.S., Reinier K., Teodorescu C., Evanado A., Kehr E., Al Samara M. et al. (2008) Epidemiology of sudden cardiac death: clinical and research implications. Prog. Cardiovasc. Dis. 51, 213–228 10.1016/j.pcad.2008.06.003 19026856 PMC2621010

[23] Lin P.H., Lee S.H., Su C.P., Wei Y.H., Damage O., Mitochondrial T.O. et al. (2003) Oxidative damage to mitochondrial DNA in atrial muscle of patients with atrial fibrillation. Free Radic. Biol. Med. 35, 1310–1318 10.1016/j.freeradbiomed.2003.07.002 14607530

[24] Tsuboi M., Hisatome I., Morisaki T., Tanaka M., Tomikura Y., Takeda S. et al. (2001) Mitochondrial DNA deletion associated with the reduction of adenine nucleotides in human atrium and atrial fibrillation. Eur. J. Clin. Invest. 31, 489–496 10.1046/j.1365-2362.2001.00844.x 11422398

[25] Bukowska A., Schild L., Keilhoff G., Hirte D., Neumann M., Gardemann A. et al. (2008) Mitochondrial dysfunction and redox signaling in atrial tachyarrhythmia. Exp. Biol. Med. 233, 558–574 10.3181/0706-RM-155 18375832

[26] Ad N., Schneider A., Khaliulin I., Borman J.B. and Schwalb H. (2005) Impaired mitochondrial response to simulated ischemic injury as a predictor of the development of atrial fibrillation after cardiac surgery: In vitro study in human myocardium. J. Thorac. Cardiovasc. Surg. 129, 41–45 10.1016/j.jtcvs.2004.03.058 15632823

[27] Kabunga P., Lau A.K., Phan K., Puranik R., Liang C., Davis R.L. et al. (2015) Systematic review of cardiac electrical disease in Kearns-Sayre syndrome and mitochondrial cytopathy. Int. J. Cardiol. 181, 303–310 10.1016/j.ijcard.2014.12.038 25540845

[28] Akar F.G. and O’Rourke B. (2011) Mitochondria are sources of metabolic sink and arrhythmias. Pharmacol. Ther. 131, 287–294 10.1016/j.pharmthera.2011.04.005 21513732 PMC3138548

[29] Akar F.G., Aon M.A., Tomaselli G.F. and O’Rourke B. (2005) The mitochondrial origin of postischemic arrhythmias. J. Clin. Invest. 115, 3527–3535 10.1172/JCI25371 16284648 PMC1280968

[30] Brown D.A., Aon M.A., Frasier C.R., Sloan R.C., Maloney A.H., Anderson E.J. et al. (2010) Cardiac arrhythmias induced by glutathione oxidation can be inhibited by preventing mitochondrial depolarization. J. Mol. Cell Cardiol. 48, 673–679 10.1016/j.yjmcc.2009.11.011 19962380 PMC2837795

[31] Morillo C.A., Klein G.J., Jones D.L. and Guiraudon C.M. (1995) Chronic rapid atrial pacing: Structural, functional, and electrophysiological characteristics of a new model of sustained atrial fibrillation. Circulation 91, 1588–1595 10.1161/01.CIR.91.5.1588 7867201

[32] Ausma J., Wijffels M., Thoné F., Wouters L., Allessie M. and Borgers M. (1997) Structural changes of atrial myocardium due to sustained atrial fibrillation in the goat. Circulation 96, 3157–3163 10.1161/01.CIR.96.9.3157 9386188

[33] Villena J.A. (2015) New insights into PGC-1 coactivators: redefining their role in the regulation of mitochondrial function and beyond. FEBS J. 282, 647–672 10.1111/febs.13175 25495651

[34] Lelliott C.J., Medina-Gomez G., Petrovic N., Kis A., Feldmann H.M., Bjursell M. et al. (2006) Ablation of PGC-1beta results in defective mitochondrial activity, thermogenesis, hepatic function, and cardiac performance. PLoS Biol. 4, e369, 10.1371/journal.pbio.0040369 17090215 PMC1634886

[35] Gurung I., Medina-Gomez G., Kis A., Baker M., Velagapudi V., Neogi S.G. et al. (2011) Deletion of the metabolic transcriptional coactivator PGC1β induces cardiac arrhythmia. Cardiovasc. Res. 92, 29–38 10.1093/cvr/cvr155 21632884 PMC3172981

[36] Ahmad S., Valli H., Edling C.E., Grace A.A., Jeevaratnam K. and Huang C.L.H. (2017) Effects of ageing on pro-arrhythmic ventricular phenotypes in incrementally paced murine Pgc-1β−/− hearts. Pflugers Arch. Eur. J. Physiol. 469, 1579–1590, 10.1007/s00424-017-2054-3 28821956 PMC5691113

[37] Valli H., Ahmad S., Fraser J.A., Jeevaratnam K. and Huang C.L.H. (2017) Pro-arrhythmic atrial phenotypes in incrementally paced murine Pgc1β−/− hearts: effects of age. Exp. Physiol. 102, 1619–1634 10.1113/EP086589 28960529 PMC5725712

[38] Valli H., Ahmad S., Chadda K.R., Al-Hadithi A.B.A.K., Grace A.A., Jeevaratnam K. et al. (2017) Age-dependent atrial arrhythmic phenotype secondary to mitochondrial dysfunction in Pgc-1β deficient murine hearts. Mech. Ageing Dev. 167, 30–45 10.1016/j.mad.2017.09.002 28919427 PMC5652526

[39] Ahmad S., Valli H., Salvage S.C., Grace A.A., Jeevaratnam K. and Huang C.L.H.L.-H. (2018) Age-dependent electrocardiographic changes in Pgc-1β deficient murine hearts. Clin. Exp. Pharmacol. Physiol. 45, 174–186 10.1111/1440-1681.12863 28949414 PMC5814877

[40] Valli H., Ahmad S., Jiang A.Y., Smyth R., Jeevaratnam K., Matthews H.R. et al. (2018) Cardiomyocyte ionic currents in intact young and aged murine Pgc-1β−/− atrial preparations. Mech. Ageing Dev. 169, 1–9 10.1016/j.mad.2017.11.016 29197478 PMC5846848

[41] Ahmad S., Valli H., Chadda K.R., Cranley J., Jeevaratnam K. and Huang C.L.H. (2018) Ventricular pro-arrhythmic phenotype, arrhythmic substrate, ageing and mitochondrial dysfunction in peroxisome proliferator activated receptor-γ coactivator-1β deficient (Pgc-1β−/−) murine hearts. Mech. Ageing Dev. 173, 92–103 10.1016/j.mad.2018.05.004 29763629 PMC6004599

[42] King J.H., Huang C.L.-H. and Fraser J.A. (2013) Determinants of myocardial conduction velocity: implications for arrhythmogenesis. Front. Physiol. 4, 1–14 23825462 10.3389/fphys.2013.00154 PMC3695374

[43] Beauchamp P., Yamada K.A., Baertschi A.J., Green K., Kanter E.M., Saffitz J.E. et al. (2006) Relative contributions of connexins 40 and 43 to atrial impulse propagation in synthetic strands of neonatal and fetal murine cardiomyocytes. Circ. Res. 99, 1216–1224 10.1161/01.RES.0000250607.34498.b4 17053190

[44] Kanagaratnam P., Rothery S., Patel P., Severs N.J. and Peters N.S. (2002) Relative expression of immunolocalized connexins 40 and 43 correlates with human atrial conduction properties. J. Am. Coll. Cardiol. 39, 116–123 10.1016/S0735-1097(01)01710-7 11755296

[45] Kurien B.T. and Hal Scofield R. (2015) Western blotting: an introduction. Methods Mol. Biol. 1312, 17–30 10.1007/978-1-4939-2694-7_5 26043986 PMC7304528

[46] Smith P.K., Krohn R.I., Hermanson G.T., Mallia A.K., Gartner F.H., Provenzano M.D. et al. (1985) Measurement of protein using bicinchoninic acid. Anal. Biochem. 150, 76–85 10.1016/0003-2697(85)90442-7 3843705

[47] Walker J.M. (1996) The bicinchoninic acid (BCA) assay for protein quantitation. The Protein Protocols Handbook 11–14 Humana Press, Totowa, New Jersey

[48] Jeevaratnam K., Poh Tee S., Zhang Y., Rewbury R., Guzadhur L., Duehmke R. et al. (2011) Delayed conduction and its implications in murine Scn5a+/- hearts: independent and interacting effects of genotype, age, and sex. Pflugers Arch. Eur. J. Physiol. 461, 29–44 10.1007/s00424-010-0906-1 21127902 PMC3016216

[49] Sabir I.N., Killeen M.J., Grace A.A. and Huang C.L.H. (2008) Ventricular arrhythmogenesis: insights from murine models. Prog. Biophys. Mol. Biol. 98, 208–218 10.1016/j.pbiomolbio.2008.10.011 19041335

[50] Huang CL-H. (2016) Murine electrophysiological models of cardiac arrhythmogenesis. Physiol. Rev. 97, 283–409 10.1152/physrev.00007.2016 PMC5539373 27974512

[51] Thomas S.A., Schuessler R.B., Berul C.I., Beardslee M.A., Beyer E.C., Mendelsohn M.E. et al. (2012) Disparate effects of deficient expression of Connexin43 on atrial and ventricular conduction. Circulation 97, 686–691 10.1161/01.CIR.97.7.686 9495305

[52] Grivennikova V.G., Kareyeva A.V. and Vinogradov A.D. (2010) What are the sources of hydrogen peroxide production by heart mitochondria? Biochim. Biophys. Acta 1797, 939–944 10.1016/j.bbabio.2010.02.013 20170624 PMC2891298

[53] Liu M., Liu H. and Dudley S.C. (2010) Reactive oxygen species originating from mitochondria regulate the cardiac sodium channel. Circ. Res. 107, 967–974 10.1161/CIRCRESAHA.110.220673 20724705 PMC2955818

[54] Bovo E., Lipsius S.L. and Zima A.V. (2012) Reactive oxygen species contribute to the development of arrhythmogenic Ca^2+^ waves during β-adrenergic receptor stimulation in rabbit cardiomyocytes. J. Physiol. 590, 3291–3304 10.1113/jphysiol.2012.230748 22586224 PMC3459043

[55] Brown D.A. and O’Rourke B. (2010) Cardiac mitochondria and arrhythmias. Cardiovasc. Res. 88, 241–249 10.1093/cvr/cvq231 20621924 PMC2980943

[56] Nishijima Y., De Blanco E.C., Khanna S., Sen C.K., Cardounel A.J., Carnes C.A. et al. (2008) Redox modification of ryanodine receptors contributes to sarcoplasmic reticulum Ca 2 leak in chronic. Circ. Res. 103, 1466–1472 10.1161/CIRCRESAHA.108.184457 19008475 PMC3274754

[57] Glass D.B., Lundquist L.J., Katz B.M. and Walsh D.A. (1989) Protein kinase inhibitor-(6-22)-amide peptide analogs with standard and nonstandard amino acid substitutions for phenylalanine 10. Inhibition of cAMP-dependent protein kinase. J. Biol. Chem. 264, 14579–14584 2760075

[58] Liu M., Sanyal S., Gao G., Gurung I.S., Zhu X., Gaconnet G. et al. (2009) Cardiac Na+ current regulation by pyridine nucleotides. Circ. Res. 105, 737–745 10.1161/CIRCRESAHA.109.197277 19745168 PMC2773656

[59] King J.H., Wickramarachchi C., Kua K., Du Y., Jeevaratnam K., Matthews H.R. et al. (2013) Loss of Nav1.5 expression and function in murine atria containing the RyR2-P2328S gain-of-function mutation. Cardiovasc. Res. 99, 751–759 10.1093/cvr/cvt141 23723061

[60] Ning F., Luo L., Ahmad S., Valli H., Jeevaratnam K., Wang T. et al. (2016) The RyR2-P2328S mutation downregulates Nav1.5 producing arrhythmic substrate in murine ventricles. Pflugers Arch. Eur. J. Physiol. 468, 655–665 10.1007/s00424-015-1750-0 26545784 PMC4792352

[61] Hothi S.S., Gurung I.S., Heathcote J.C., Zhang Y., Booth S.W., Skepper J.N. et al. (2008) Epac activation, altered calcium homeostasis and ventricular arrhythmogenesis in the murine heart. Pflugers Arch. Eur. J. Physiol. 457, 253–270 10.1007/s00424-008-0508-3 18600344 PMC3714550

[62] Valli H., Ahmad S., Sriharan S., Dean L.D., Grace A.A., Jeevaratnam K. et al. (2018) Epac-induced ryanodine receptor type 2 activation inhibits sodium currents in atrial and ventricular murine cardiomyocytes. Clin. Exp. Pharmacol. Physiol. 45, 278–292 10.1111/1440-1681.12870 29027245 PMC5814738

[63] Maurer P. and Weingart R. (1987) Cell pairs isolated from adult guinea pig and rat hearts: effects of [Ca2+]i on nexal membrane resistance. Pflügers Arch. Eur. J. Physiol. 409, 394–402 10.1007/BF00583793 3627957

[64] Noma A. and Tsuboi N. (1987) Dependence of junctional conductance on proton, calcium and magnesium ions in cardiac paired cells of guinea-pig. J. Physiol. 382, 193–211 10.1113/jphysiol.1987.sp016363 2442361 PMC1183020

[65] Garcia-Dorado D., Ruiz-Meana M., Padilla F., Rodriguez-Sinovas A. and Mirabet M. (2002) Gap junction-mediated intercellular communication in ischemic preconditioning. Cardiovasc. Res. 55, 456–465 10.1016/S0008-6363(02)00441-8 12160942

[66] Jabr R.I., Hatch F.S., Salvage S.C., Orlowski A., Lampe P.D. and Fry C.H. (2016) Regulation of gap junction conductance by calcineurin through Cx43 phosphorylation: implications for action potential conduction. Pflugers Arch. Eur. J. Physiol. 468, 1945–1955 10.1007/s00424-016-1885-7 27757582 PMC5138272

[67] Jeong E.-M., Liu M., Sturdy M., Gao G., Varghese S.T., Sovari A.A. et al. (2012) Metabolic stress, reactive oxygen species, and arrhythmia. J. Mol. Cell Cardiol. 52, 454–463 10.1016/j.yjmcc.2011.09.018 21978629 PMC3264827

[68] Yang K.-C., Kyle J.W., Makielski J.C. and Dudley S.C. (2015) Mechanisms of sudden cardiac death: oxidants and metabolism. Circ. Res. 116, 1937–1955 10.1161/CIRCRESAHA.116.304691 26044249 PMC4458707

[69] Yang K.C., Bonini M.G. and Dudley S.C. (2014) Mitochondria and arrhythmias. Free Radic. Biol. Med. 71, 351–361 10.1016/j.freeradbiomed.2014.03.033 24713422 PMC4096785

[70] Edling C.E., Fazmin I.T., Chadda K.R., Ahmad S., Valli H., Huang CL-H et al. (2019) Atrial Transcriptional Profiles of Molecular Targets Mediating Electrophysiological Function in Aging and Pgc-1β Deficient Murine Hearts. Front Physiol. 10, 497 10.3389/fphys.2019.00497 31068841 PMC6491872

[71] Verheule S., Van Batenburg CAJAC, Coenjaerts F.E.J., Kirchhoff S., Willecke K. and Jongsma H.J. (1999) Cardiac conduction abnormalities in mice lacking the gap junction protein connexin40. J. Cardiovasc. Electrophysiol. 10, 1380–1389 10.1111/j.1540-8167.1999.tb00194.x 10515563

[72] Edling C.E., Fazmin I.T., Chadda K.R., Ahmad S., Valli H., Grace A.A. et al. (2019) Ageing in Pgc-1β-/- mice modelling mitochondrial dysfunction induces differential expression of a range of genes regulating ventricular electrophysiology. Biosci. Rep. 39, BSR20190127 10.1042/BSR20190127 30914453 PMC6470410

